# Cholera at New York

**Published:** 1888-02

**Authors:** 


### CHOLERA AT NEW YORK.

Within the last sixty years the United States have suffered from five epidemics of cholera, which have uniformly followed the prevalence of the disease in Europe, namely, in 1832, 1849, 1854, 1866, and 1873. In each instance the disease was brought by immigrants from Europe. The arrival of the French steamship *Alesia* at New York, on September 23 last, with cases of cholera on board, was therefore sufficient to cause anxiety to some people as to the possibility of the infection reaching the shore. The *Alesia* sailed from Marseilles on August 30, and arrived at Naples on September 1. There she took on board about 300 emigrants and 3 cabin passengers, making her total complement 609 persons, exclusive of the crew of 45 men. The vessel left Naples on September 3, and until September 12 all appears to have gone well. Then, however, cholera unmistakably broke out amongst the Italians who had been shipped at Naples, and before the vessel reached American waters six persons (including two of the crew) had died from the disease, and had been buried at sea, and a number of other cases had occurred. One of the deaths ensued within eighteen hours after attack, whilst the illness of none of the fatal cases exceeded three days. On arrival at New York the vessel was taken in hand by Dr. Smith, the health officer, who vigorously applied the principles of isolation, sequestration, and disinfection, relied upon in America for preventing the spread of disease from shipping. Some sixteen patients were at once removed to the hospital on Swinburne Island, and a number of other passengers, who had been exposed to infection, were kept under observation at Hoffman's Island. The vessel itself appears to have been in a dirty condition, and was forthwith cleansed and disinfected. Since the arrival of the vessel at New York twenty additional deaths from cholera and a number of further cases have occurred amongst the passengers.

Dr. Smith has expressed an unhesitating opinion that there is no danger of any spread of the disease on shore. The precautions against the entrance of cholera have been constant ever since the disease began its ravages in Italy. The passenger list of every vessel that arrives is watched, and all immigrants from infected parts are carefully inspected. Even when the immigrants themselves show no signs of disease, their baggage is thoroughly fumigated. The facilities for caring for the sick are good, and the accommodation for the "quarantined" passenger is satisfactory. There are some people in New York, however, who are apprehensive that cholera may appear in the city next spring. It will be seen that quarantine as practiced in many European countries is not now followed in the United States, but that the preferable system of medical inspection of ships is in vogue.

Good sanitation on shore and watchful care to secure the isolation and disinfection of the first cases of infectious disease are the principles relied upon. Although the term "quarantine" is now in common use in connection with the preventive measures adopted at New York and other American ports, its use does not imply detention of vessels for any specified time, but only for such time as is needful to determine the presence or absence of infection, and to effect, if present, its destruction. The removal to hospital of infected passengers or members of the crew, and of others to proper quarters, is provided for, as well as the careful disinfection of clothing, baggage, and cargo. But there seems to be some local dissatisfaction at New York respecting the scantiness of the sums voted by Congress for securing the completely efficient carrying out of this programme. If there is any substantial ground for such dissatisfaction it is regrettable. The last occasion on which cholera was brought into New York waters was in September and October, 1873, when four steamers -- one from Hamburg — arrived, having cases of sickness on board. The disease then, unfortunately, reached the mainland.—*British Medical Journal.*

